# Health-related quality of life and *Chlamydia trachomatis* infection in sexually experienced female inner-city students: a community-based cross-sectional study

**DOI:** 10.1177/0956462416650095

**Published:** 2016-07-10

**Authors:** Trisha Sri, Eleanor Southgate, Sarah R Kerry, Claire Nightingale, Pippa Oakeshott

**Affiliations:** 1 Population Health Research Institute, St George’s University of London, London, UK; 2 Queen Mary University of London, Centre for Primary Care and Public Health, London, UK

**Keywords:** Health-related quality of life, EQ-5D, cross-sectional study, *Chlamydia trachomatis*, women’s health

## Abstract

This cross-sectional study was undertaken to compare health-related quality of life (EQ-5D) in women with and without undiagnosed *Chlamydia trachomatis* infection. We analysed data from 2401 multi-ethnic sexually active female students aged 16–27 years who were recruited to a randomised controlled trial of chlamydia screening – the prevention of pelvic infection trial in 2004–2006. At recruitment, all participants were asked to provide self-taken vaginal swabs for chlamydia testing and to complete a sexual health questionnaire including quality of life (EQ-5D). Most women (69%) had an EQ-5D of one representing ‘perfect health’ in the five dimensions: mobility, self-care, usual activities, pain/discomfort and anxiety/depression. We therefore compared the proportion of women with an EQ-5D score < 1 implying ‘less than perfect health’ in women with and without chlamydia infection, and women with symptomatic chlamydia versus the remainder. The proportion of women with EQ-5D score < 1 was similar in women with and without undiagnosed chlamydia: 34% (47/138) versus 31% (697/2263; RR 1.11, 95% CI 0.87 to 1.41). However, more women with symptomatic chlamydia had EQ-5D score < 1 than the remainder: 45% (25/55) versus 31% (714/2319; RR 1.47, CI 1.10 to 1.98). In this community-based study, EQ-5D scores were similar in women with and without undiagnosed chlamydia. However, a higher proportion of women with symptomatic chlamydia infection had ‘less than perfect health’. Undiagnosed chlamydia infection may not have a major short-term effect on health-related quality of life, but EQ-5D may not be the best tool to measure it in this group.

## Introduction

The 2014 European Centres for Disease Prevention and Control report called for research on ‘the impact of chlamydia infection on quality of life to help to provide more accurate assessments of the cost-effectiveness of chlamydia screening’.^1^ Most people with chlamydia infection have few symptoms, and the effect on health-related quality of life in the short term is unclear. EQ-5D scores in people with and without chlamydia might be useful for exploring the health cost of chlamydia and whether screening is worthwhile. It could also inform wider debates around the impact of chlamydia on quality of life. To date, there have been no UK studies looking at health-related quality of life associated with chlamydia in sexually active, young, multi-ethnic women recruited outside healthcare facilities.

We have baseline cross-sectional EQ-5D scores, sexual health questionnaires and chlamydia test results from 2401 women who took part in the prevention of pelvic infection (POPI) chlamydia screening trial.^2,3^ Our aims were:

1. To compare EQ-5D scores in women with and without undiagnosed chlamydia.
2. To compare EQ-5D scores in women with symptomatic chlamydia versus the remainder.

## Methods

### Data collection

Details of the POPI trial have been published elsewhere.^2^ Briefly, during 2004–2006, a multi-ethnic sample of 2529 16–27-year-old, sexually active female students were recruited from 20 universities and further education colleges in London to a randomised controlled trial to determine whether screening for chlamydia reduced the incidence of pelvic inflammatory disease (PID) over the following 12 months. Students who had never had sexual intercourse and those who had been tested for chlamydia in the past three months were excluded from the trial.

### Baseline assessment

At baseline, participants were asked to complete a questionnaire on sexual health and quality of life (EQ5-D and EQ-VAS), and to provide self-taken vaginal swabs to test for *Chlamydia trachomatis*. Participants randomly allocated to the intervention group had their swabs tested for chlamydia in the next week, and those with positive tests were referred for treatment. Samples from participants allocated to the deferred screening group were frozen at −80°C and were tested after 12 months.^2^ The sexual health questionnaire was developed by the researchers and included questions on demographics, number of sexual partners and on four symptoms that may be associated with PID: pelvic discomfort, pain during sexual intercourse abnormal vaginal discharge and bleeding between periods.^2^ Women who reported all four of these symptoms were counted as ‘symptomatic’ in the analysis.

### Health-related quality of life (EQ-5D)

Questionnaires assessed health-related quality of life using the EQ-5D tool. This is the preferred method of utility measurement by the UK National Institute for Health and Clinical Excellence^3^ and is generic rather than disease specific. The EQ-5D has five dimensions: mobility, self-care, usual activities, pain/discomfort and anxiety/depression. Weighted UK preference values are linked to the self-reported health state scores for a 0–1 index, where 0 is death and 1 is perfect health.

### Sample size

**Figure 1.**
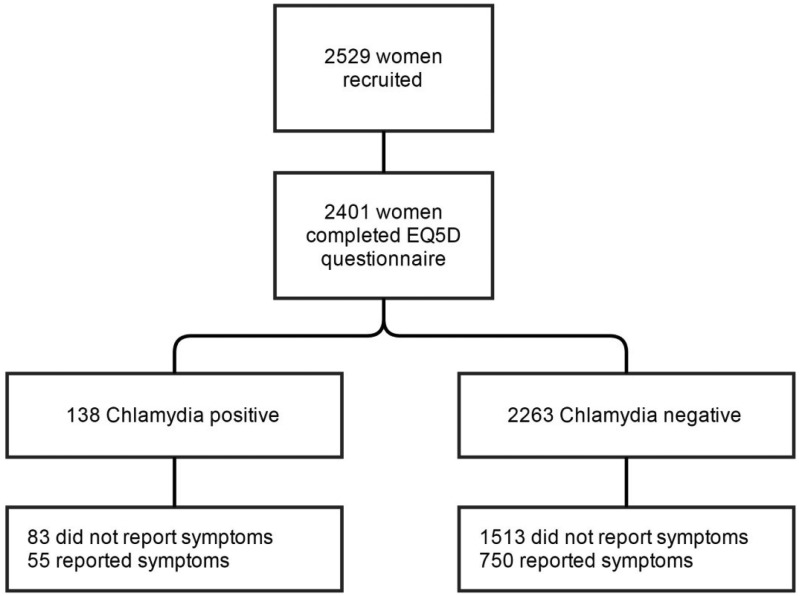
Flow chart illustrating the number of participants throughout the study.

We were limited to the 2529 female students recruited to the POPI trial^2^ (Figure 1). Of these, 2401 (95%) answered questions on health-related quality of life at baseline and provided samples suitable for chlamydia testing and were included in the cross-sectional study. (The questionnaire comprised an A4 sheet with questions on lifestyle on the first page and the EQ-5D on the second page. It is likely that many of the 128 participants who did not complete EQ-5D did not turn over the questionnaire before sealing it in the envelope.)

### Statistical analyses

Most participants (69%, 1657/2410) had no problems with any of the five dimensions and scored 1 implying ‘perfect health’. We therefore compared the proportion with EQ-5D index score < 1 (implying ‘less than perfect health’ in one or more of the five dimensions) in women with and without chlamydia infection using relative risks. We then compared this measure in women with symptomatic chlamydia versus the remainder. Mean EQ-5D scores were also estimated.

## Results

### Sample demographics

Of the 2401 women included in the analysis, 68% were recruited from universities and the remainder from further education (FE) colleges; 27% were of black ethnicity, 44% were aged less than 20 years and 32% were smokers. With regard to sexual health at baseline, 41% reported two or more sexual partners in the preceding 12 months, 30% were aged under 16 at sexual debut, and 11% reported having an STI in the preceding year. Forty six per cent reported that they did not use condoms.

### Health-related quality of life scores and chlamydial infection

Table 1 shows that the proportion of women with EQ-5D score < 1 was similar in women with and without undiagnosed chlamydia: 34% versus 31%. However, more women with symptomatic chlamydia had EQ-5D score < 1 than the remainder: 45% versus 31% (*p* = 0.02). (Results were similar if 81 asymptomatic chlamydia positives who provided details on symptoms were excluded from the ‘remainder’ group.) Mean EQ-5D scores were in line with these findings: 0.88 in those with symptomatic chlamydia versus 0.95 in the remainder (*p* < 0.01).

**Table 1.** EQ-5D scores in 2401 women with and without undiagnosed chlamydia infection, and in women with symptomatic chlamydia infection versus the remainder.

	EQ-5D < 1 Less than perfect health	EQ-5D = 1 Perfect health	Relative risk	*p*
Chlamydia positive n = 138 (mean EQ-5D .93)	47 (34%)	91	1.11 (0.87 to 1.41)	0.42
Chlamydia negative n = 2263 (mean EQ-5D .93)	697 (31%)	1566		
Symptomatic^a^ chlamydia infection n = 55 (mean EQ-5D .88)^b^	25 (45%)	30	1.47 (1.10 to 1.98)	0.02
No chlamydia or asymptomatic chlamydia n = 2319 (mean EQ-5D .95)^b^	714 (31%)	1605		

a Two women with chlamydia and 25 without chlamydia did not answer the question on symptoms.

b *p* < 0.01 Wilcoxon rank sum test.

## Discussion

### Principal findings

In this community-based study, EQ-5D scores were similar in women with and without undiagnosed chlamydia. However, a higher proportion of women with symptomatic chlamydia had EQ-5D score < 1 than the remainder.

### Strengths and weaknesses

To our knowledge, this is the first UK study of health-related quality of life and chlamydia infection in sexually active, young, multi-ethnic female students recruited from a community setting.^1^ It may be unique in that women did not know whether or not they had chlamydia at the time of completing the EQ-5D. Findings may help to inform wider debates around the impact of chlamydia on quality of life.

The main limitation is that the EQ-5D was not completed at time of chlamydia diagnosis. Since participants did not know if they had chlamydia, the findings can only relate to symptoms and not to the implications of a positive diagnosis with the possible associated effect on relationships, stigma and anxiety regarding infertility.^4,5^ In addition, we did not assess the effect on quality of life of long-term potential complications of chlamydia such as PID, chronic pelvic pain, tubal factor infertility or ectopic pregnancy. The EQ5-D may be less suitable for a young population who are unlikely to have problems in three of the five domains: mobility, self-care and usual activities. We therefore divided participants into those EQ-5D < 1 versus the remainder. This approach is novel and might stimulate debate. However, results were similar using the standard method of EQ-5D analysis. We did not use data from the EQ-VAS visual analogue scale as these were skewed. Findings may not be generalisable and may not apply to other groups such as men in whom chlamydia infection may be more likely to cause symptoms. Although the study was completed almost ten years ago, this is one of the few available with data on STIs and EQ-5D.^3^ Finally, these results only allow us to comment on associations – we cannot infer any causality from reported EQ-5D scores.

### Comparison with other studies

A previous analysis in the same cohort showed that women who developed pelvic infection had lower quality of life scores than those who did not at both baseline and follow-up. However, pelvic infection did not appear to reduce EQ-5D scores further.^3^ By contrast, most previous studies of the psychological or physical impact of chlamydia infection have been qualitative and have focused on participants with a current or recent diagnosis of chlamydia. Duncan et al.^4^ found that women had three main concerns: stigma surrounding the infection, anxiety about future fertility and apprehension about their partner’s reaction. Interviews with participants in the CLASS population-based screening studies showed similar findings.^5^ Interestingly, no one regretted being screened for chlamydia. However, there appears to be a paucity of studies on quality of life related to sexual health in a student population.^1^

Finally, some studies^6^ have used health utility weights for chlamydia based on the 1999 Institute of Medicine report.^7^ Models simulating the natural history of chlamydia may be combined with cost and utility data to estimate the number of quality-adjusted life-years (QALYs) associated with chlamydia. Economic models estimate the QALYs lost if chlamydia is untreated, in order to estimate the QALY gains associated with treating/preventing chlamydia. Crucially, these models include potential long-term sequelae such as tubal infertility. By contrast, our study focused only on short-term health-related quality of life.

### Implications

Having asymptomatic undiagnosed chlamydia did not seem to affect EQ-5D scores in the short term in this group of young women, although symptomatic chlamydia was associated with ‘less than perfect’ quality of life. However, the EQ-5D may not be the best or most sensitive measure of health-related quality of life in a young sexually active female population. Issues around choosing between condition-specific measures and generic instruments need further investigation.

## Key messages

- EQ-5D scores were similar in women with and without undiagnosed chlamydia infection.
- Women with symptomatic chlamydia infection were more likely than the remainder to have EQ-5D score < 1 implying ‘less than perfect health’.
- It is possible that undiagnosed chlamydia infection does not have a major effect on health-related quality of life in young women in the short term.
- However, the EQ-5D may not be the best tool to measure health-related quality of life in sexually active young women.
